# Deficiency in Lipoteichoic Acid Synthesis Causes a Failure in Executing the Colony Developmental Program in *Bacillus subtilis*

**DOI:** 10.3389/fmicb.2017.01991

**Published:** 2017-10-24

**Authors:** Gideon Mamou, Osher Fiyaksel, Lior Sinai, Sigal Ben-Yehuda

**Affiliations:** Department of Microbiology and Molecular Genetics, Institute for Medical Research Israel-Canada, The Hebrew University Hadassah Medical School, Hebrew University of Jerusalem, Jerusalem, Israel

**Keywords:** *Bacillus subtilis*, lipoteichoic acid, bacterial colony, bacterial multicellularity, Bacterial cell biology

## Abstract

Colonies are an abundant form of bacterial multicellularity; however, relatively little is known about the initial stages of their construction. We have previously described that colony development of the soil bacterium *Bacillus subtilis* is a highly ordered process, typically initiating with the formation of extending cell chains arranged in a Y shape structure. Furthermore, we demonstrated that Y arm extension is a key for defining the size of the future colony. Here we conducted a genetic screen surveying for mutants deficient in these early developmental stages, and revealed LtaS, the major lipoteichoic acid (LTA) synthase, to be crucial for execution of these events. We found that the ltaS mutant fails to produce proper Y shape structures, forming extremely elongated chains of cells with no evidence of chain breakage, necessary for Y shape formation. Furthermore, we show that frequent cell death at the tips of the cell chains is a major cause in limiting arm extension. Collectively, these perturbations lead to the production of a small sized colony by the mutant. Thus, deficiency in LTA synthesis causes a mechanical failure in executing the colony developmental program.

## Introduction

Bacteria are highly adaptable organisms capable of living in a vast variety of niches, either as planktonic single cells, or in large bacterial communities. Accumulating evidence highlights that living in large bacterial assemblies is beneficial for exploiting a wide variety of nutrients and withstanding harsh environmental conditions, such as antibiotic assault (Gilbert and Mcbain, 2001; Lewis, 2001; Stickler, 2002; Anderson and O'toole, 2008). Bacterial assemblies provide shelter and a steadier environment, conditions that are rarely achieved during the bacterium planktonic life. In addition, multicellularity facilitates the exchange of genetic information, which enables the acquisition of new features and consequently accelerates bacterial evolution (Shapiro, 1998; Li et al., 2001; Jakubovics et al., 2013).

Bacterial multicellularity has several forms starting from relatively simple chains of cells up to biofilms, structured macrocolonies and fruiting bodies. A key feature in various forms of bacterial multicellularity is division of labor which is achieved through differentiation into subpopulations that execute distinct activities. Cross-sectioned inspection of aging *Escherichia coli* (*E. coli*) colonies revealed that inner regions are characterized by cells producing amyloidic curli fibers, whereas the bottom zone of the macrocolonies is comprised of dividing cells encased within tight flagella mesh (Serra et al., 2013). In the soil bacterium *Bacillus subtilis* (*B. subtilis*) specific subpopulations of cells such as surfactin producers, matrix producers, motile and sporulating cells, were shown to reside in different zones within biofilms (Veening et al., 2006; Vlamakis et al., 2008; Lopez et al., 2009). Numerous studies identified genes involved in colony and biofilm formation in several different bacterial species, with most of the discovered genes being implicated in surface attachment, matrix production, and intercellular communication (e.g.,O'toole and Kolter, 1998; Pratt and Kolter, 1998; Wen and Burne, 2002; Branda et al., 2004).

**Figure 1:**
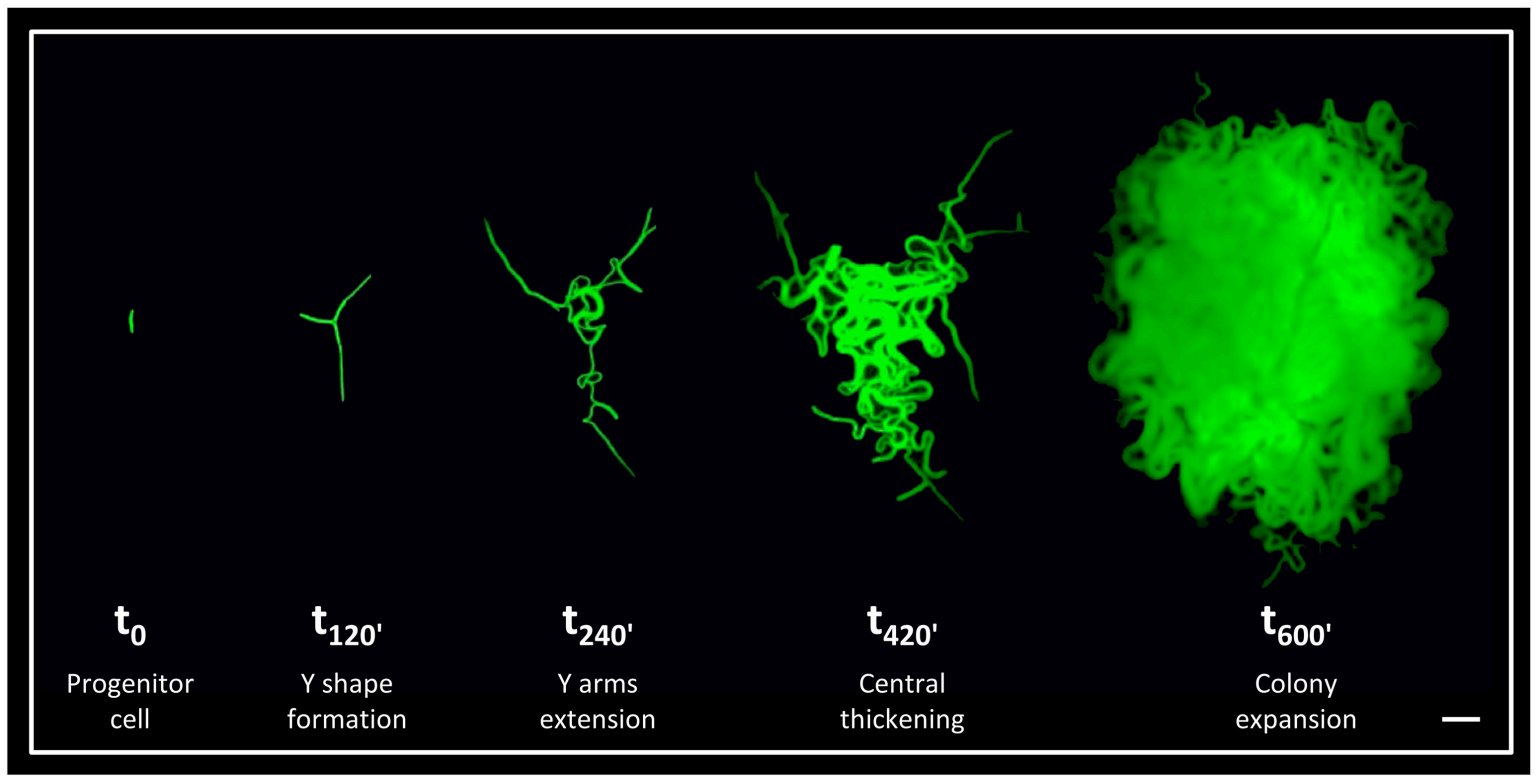
The early stages of *B. subtilis* colony development. Time lapse fluorescence images of AR16 (P_*rrnE*_*-gfp*) cells at different stages of colony development at the indicated time points (min), as previously characterized (Mamou et al., 2016). Scale bar 20 μm.

Although the architecture of bacterial colonies is well-characterized, the earliest hours in their formation is relatively unexplored. To shed light on these early events, we have previously followed developing *B. subtilis* colonies from a single progenitor cell up to a three dimensional structure at high resolution (Mamou et al., 2016). We discovered that an accurate developmental program is executed at the very first hours of colony construction (Figure 1). Initially, the progenitor cell divides to produce an elongated chain of cells, which tends to break in the middle and create a typical Y shape. Subsequently, the three arms of the Y shape rapidly extend outwards to conquer a large radial area. In the following stage, the colony thickens outwards from its center, until the major bulk of the cells reaches the borders outlined by the Y arms. In the final stage, the colony expands radially giving rise to the mature state (Figure 1). Further, by eradicating specific cells within the Y arms during colony formation, we provided evidence that the reach of the Y arms determines colony size (Mamou et al., 2016).

We have previously revealed that a mutant in the phosphodiesterase YmdB displays aberrant developmental morphology, as it was unable to produce Y shape patterns, resulting in the formation of small sized colonies (Mamou et al., 2016). Here we employed a genetic screen to uncover additional genes involved in the earliest stages of colony construction. Our analysis revealed that LtaS, the major lipoteichoic acid (LTA) synthase in *B. subtilis*, is crucial for execution of these early stages. LtaS was previously shown to incorporate polyglycerol phosphate polymers to produce membrane bound LTAs, an important component of Gram-positive cell envelope (Grundling and Schneewind, 2007). We found that *ltaS* mutant cells fail to properly form the typical Y shape, largely due to the frequent death of cells located at the tips of the arms. We propose that this accelerated death emanates from pre-mature cell aging due to the lack of LTA polymers.

## Materials and methods

### Bacterial strains and plasmids

Bacterial strains used in this study are listed in Table S1, plasmid constructions are described in Table S2 and primers are listed in Table S3.

### General methods

All general methods were carried out as described previously (Harwood and Cutting, 1990). For analysing growth in liquid medium, cultures were inoculated at an OD_600_ of 0.05 from an overnight culture, and OD_600_ was measured every 20–30 min using CO8000 Cell Density Meter (Biochrom). For colony development experiments, growth was carried out at 37°C. Cells were incubated in liquid Luria Broth (LB) medium at 23°C overnight, and diluted to cell density of ~1 cell/μl (accordingly, a typical start up culture of OD_600_ 0.7 was diluted up to 10^−5^). Isopropyl β-D-1-thiogalactopyranoside (IPTG) was added to a final concentration of 1 mM, when indicated. Mature colonies grown overnight on LB plates were observed and photographed using Discovery V20 stereoscope (Zeiss) equipped with Infinity1 camera (Luminera) and the diameter of each colony was measured using a scaled image.

### Transposon mutagenesis

Transposon mutagenesis was carried out using the mariner-derived TnYLB transposon, which generates chloramphenicol (Cm)-marked insertions in the chromosome. This transposon requires only a “TA” dinucleotide as the essential target in the recipient DNA, and thus lacks specific hot spots (Le Breton et al., 2006). The screen was conducted using strain DS8274, harboring a plasmid with the TnYLB element (Pozsgai et al., 2012). This plasmid has a temperature sensitive origin of replication, active only at low temperature (23–30°C). DS8274 was grown overnight at 23°C in liquid LB, plated on LB-Cm containing plates, and incubated at 48°C to generate transposon mutant colonies. Each colony was then transferred into a new, numbered LB-Cm plate and incubated at 30°C for 24 h. All colonies displaying abnormal colony size or morphology (~200) were further examined using a stereoscope (Discovery V20, Zeiss). Overall, 12,000 mutant colonies were screened, and 17 candidates were selected for further analysis. In order to determine the insertion site of the transposon, chromosomal DNA was isolated from the candidate strain, and 1 μg of chromosomal DNA was digested with Sau*3A*I for 1 h, heat inactivated for 20 min, and 0.1 μg of digested DNA was ligated using T4 DNA ligase at room temperature for 1 h. PCR was conducted using ligation reaction mixtures as a template, primers 2757/2729 (Table S3), and Q5 DNA polymerase (New England BioLabs). Each PCR was purified (Qiagen PCR purification kit) and sequenced using primer 2757 (Table S3). Sequences were identified using basic local alignment search tool (BLAST) (Altschul et al., 1990). Candidate genes were annotated using the information from subtilist and subtiwiki websites:

http://genolist.pasteur.fr/SubtiList
http://subtiwiki.uni-goettingen.de

### Live imaging of developing colonies

A custom designed construct was used to grow bacterial colonies under the microscope as previously described (Mamou et al., 2016). Bacterial cells were spotted at a concentration of ~1 cell/ μl on solid LB medium. When indicated, PI (Sigma) was added to a final concentration of 5 μg/ml. Construct was covered with a 35 mm cultFoil membrane (Pecon) to reduce dehydration, and incubated at 37°C in Lab-Tek S1 heating insert (Pecon) placed inside an incubator XL-LSM 710 S1 (Pecon). Developing colonies were visualized and photographed by CLSM LSM700 (Zeiss) with Plan-Aporchromat x10/NA0.45 (Zeiss). Cells expressing GFP or Dronpa were irradiated using 488 nm laser beam, while cells stained with PI were irradiated using 555 nm laser beam. For each experiment, both transmitted and reflected light were collected. System control and image processing were carried out using Zen software version 8.0 (Zeiss).

### Measuring the radial area occupied by a developing colony

Calculating the radial area occupied by a given colony was carried out by measuring the area of the minimal circle harboring the entire population of cells at the analyzed stage. The measured area was then normalized to the total number of cells in the developing colony, as estimated by measuring the total chain length. At least 10 colonies were used to estimate the average area.

### Analyzing the fraction of dead cells within developing colonies

To estimate the fraction of dead cells in the entire colony and at the tips of the extending arms we used cells expressing GFP (green), and grew them in the presence of PI (red), which labels dead cells. At the time point of interest the total number of green and red pixels (above the background noise level) was calculated using Zen software version 8.0 (Zeiss). The fraction of dead cells was calculated as the number of red pixels divided by the number of green pixels. In some cases where the phase contrast image clearly showed that cells adjacent to PI labeled cells seemed to be dying, as indicated by the loss of their green signal, these cells were also counted as dead cells. Estimating the fraction of dead cells near the tips was conducted in a similar manner. In this analysis only cells, located up to 50 μm from the tip of the arm, were examined.

### Tracking cell lineage using dronpa

For tracking cell lineages, strains carrying the *rplA-Dronpa* fusion were analyzed (Table S1). Initially, Dronpa fluorescence was bleached in all cells of the colony (488 nm laser, 5%, 20–40 iterations). Then, fluorescence was reactivated by short irradiation, only in selected cells (405 nm, laser 1%, 10–20 iterations). Since newly synthesized Dronpa in non-activated cells interfered with tracking of the activated cells, this signal was re-bleached when required (488 nm laser, 5%, 10–20 iterations). Distinguishing between the selected cell lineage and other cells in the colony was based on the difference in signal intensity.

### Statistical analysis

*P*-value and statistical significance were determined by unpaired *t*-tests and were calculated using online data analysis website (Graphpad Software): https://www.graphpad.com/quickcalcs/ttest1/?Format=C

## Results

### Transposon-based genetic screen reveals an array of mutations affecting colony size

**Figure 2:**
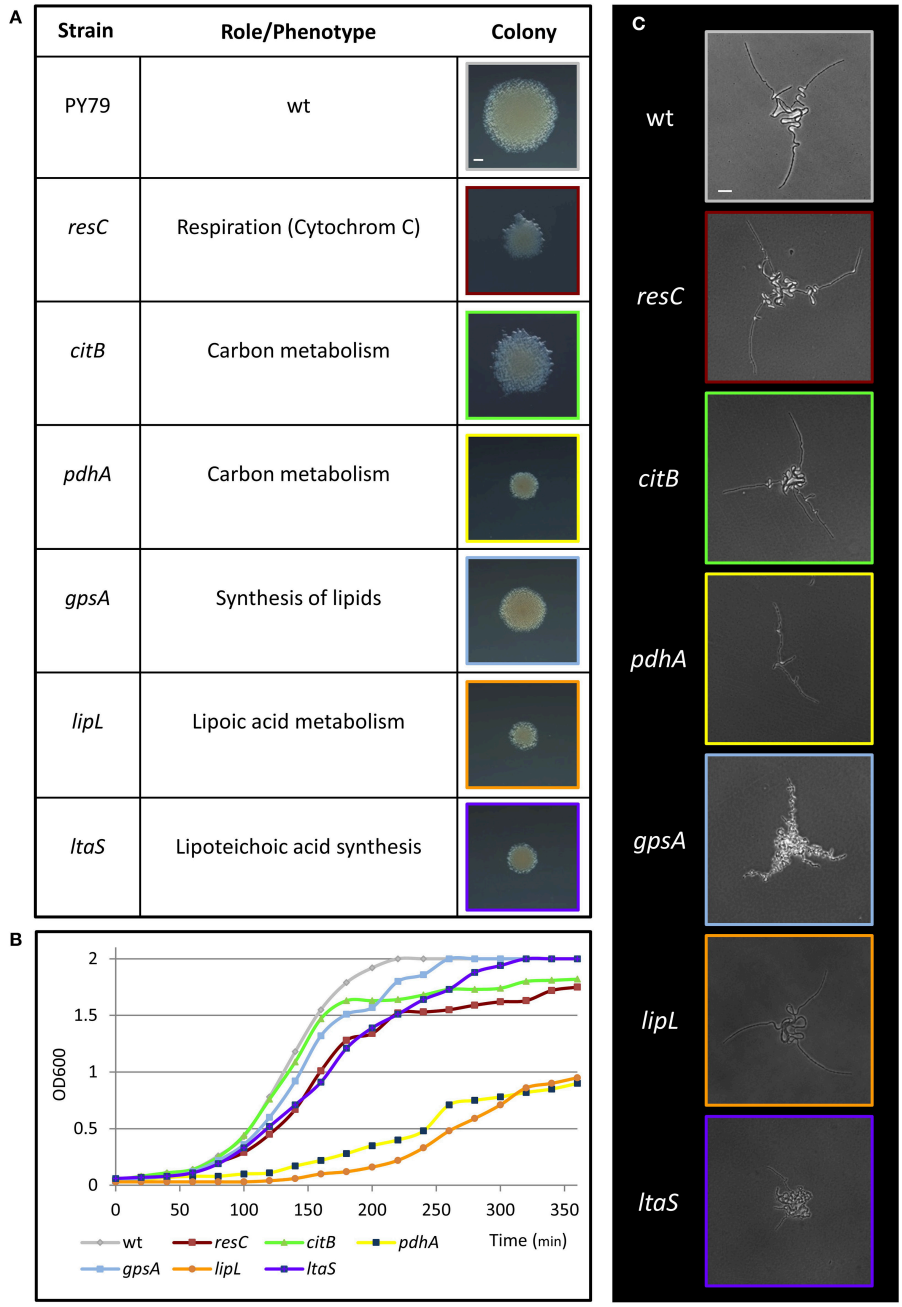
Metabolic mutants identified in the genetic screen for deficiency in colony formation. **(A)** A table presenting metabolic transposon mutant strains, which were found to produce small colonies in the transposon screen. The name and annotation (http://subtiwiki.uni-goettingen.de/) of the disrupted gene are listed. Images represent colonies of the indicated mutant colonies after 24 h of incubation on LB agar at 37°C. Scale bar 0.5 mm. **(B)** Growth curves of wt (PY79) and the indicated transposon mutant strains in liquid LB at 37°C. **(C)** Colonies of wt (PY79) and the indicated transposon mutant strains were incubated on LB agar at 37°C and followed by time lapse microscopy. Shown are developing colony images taken at ~4.5 h of growth. Scale bar 25 μm. Colors correspond to each strain throughout the figure.

To discover genes, pathways and processes required for early stages of colony formation, we performed unbiased mutagenesis using a mariner transposon (Pozsgai et al., 2012), screening for colonies with aberrant morphologies. From 12,000 visually screened colonies, 17 different mutants that produced small colonies or exhibited colonies with atypical architecture were isolated. The mutations were mapped to 17 different loci, including genes involved in metabolism and energy production, induction of stress response, basic cellular processes and intercellular interactions (Figure 2A; Figure S1A). Mutations in genes implicated in metabolism and energy production generated very small colonies that could emanate from slow growth rate or deficiencies in executing the early colony developmental program. To examine these possibilities, we followed the mutants' growth rate, concurrently with their effect on Y shape formation. The investigated mutants exhibited an array of various growth rates (Figure 2B; Figure S1B), however; only the *ltaS* mutant showed clear abnormal developmental patterns, failing to form either the typical Y shape or significantly extending arm chains (Figure 2C; Figure S2A). *ltaS* mutant displayed an extended lag phase, however its growth rate was only slightly perturbed in comparison to wild type at 37°C, but the change was more significant at lower temperatures (Figure S2B). Thus, the ability to form the characteristic Y shape is independent of growth rate.

### Lipotechoic acid synthesis plays an essential role in colony development

**Figure 3:**
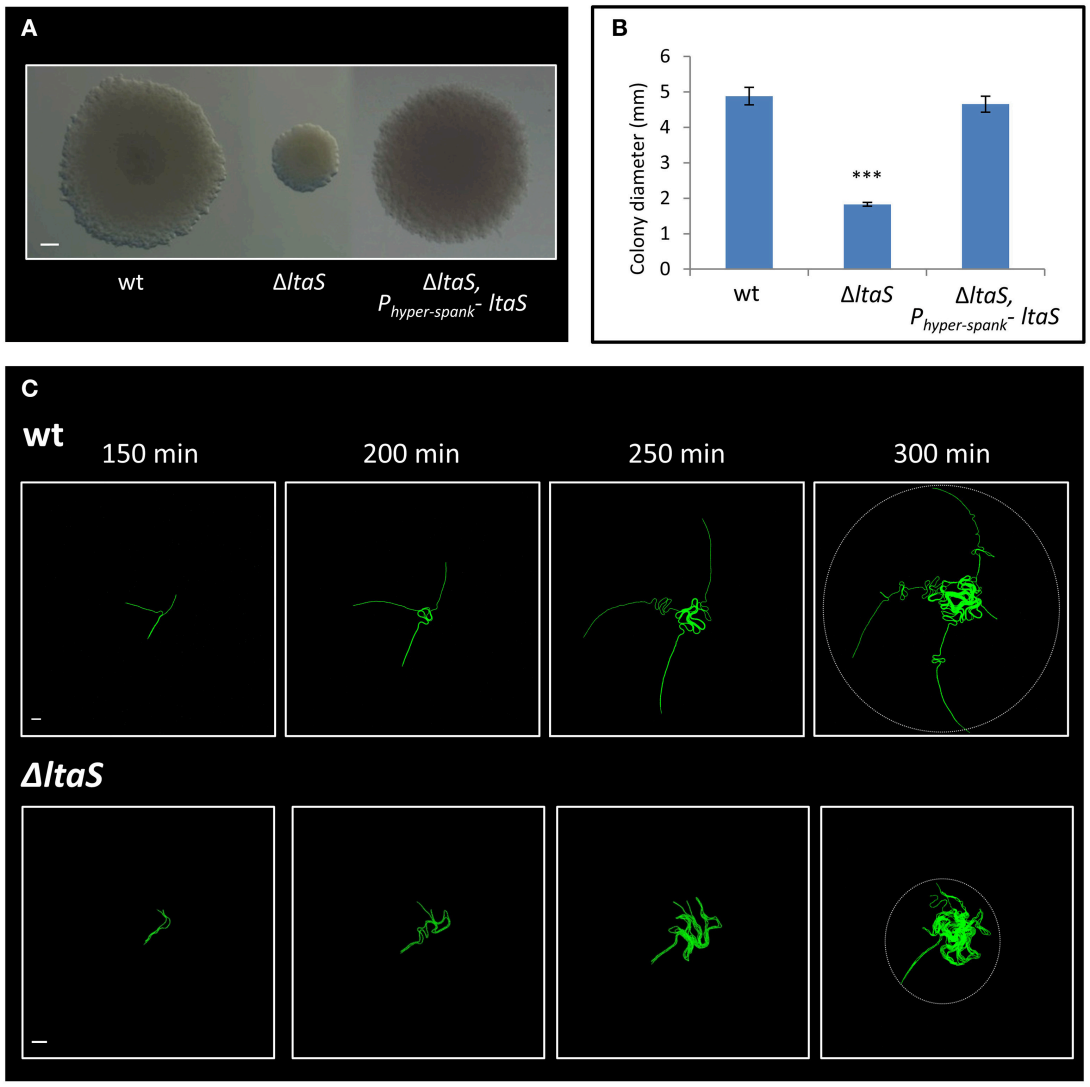
LtaS is essential for the early stages of colony development. **(A)** Cells of PY79 (wt), GM21 (Δ*ltaS*) and GM45 (Δ*ltaS, P*_*hyper*−*spank*_*- ltaS*) were grown on solid LB medium and incubated at 37°C for 20 h. Shown are typical colonies photographed using a binocular. Scale bar 0.5 mm. **(B)** The average diameter of colonies described in **(A)**. At least 15 colonies were measured for each strain. Statistical significance was calculated using unpaired *t*-test. **(C)** Time lapse fluorescence images of wt AR16 (P_*rrnE*_*-gfp*) (upper) and GM24 (Δ*ltaS*, P_*rrnE*_*-gfp*) (lower) developing colonies followed by CLSM at the indicated time points. Dashed circle at *t* = 300 min marks the radial area occupied by each developing colony. Scale bars 10 μm. ^***^P < 0.001, Very significant.

The *ltaS* gene encodes for the major LTA synthase, which is essential for proper cell wall synthesis. LTAs are anionic glycerolphosphate polymers, anchored to the cell membrane (Grundling and Schneewind, 2007; Schirner et al., 2009). Constructing an *ltaS* knockout strain recapitulated the small sized colony observed with the transposon insertion, a phenotype that was complemented by ectopic expression of the wild type gene (Figures 3A,B). Previous studies have shown that mutating *ltaS* leads to defects in cell separation and proper chain breakage (Schirner et al., 2009; Kiriyama et al., 2014). We reasoned that such defects could impact early colony developmental stages. To address this issue, the Δ*ltaS* colony was closely investigated by time lapse microscopy. The mutant cells failed to extend away from colony center, and after a short time the chains appeared to curl and create a dense bulk of coiled chains, with no evident chain breakage (Figure 3C; Movies S1, S2). The average radial area occupied by colony founders of the Δ*ltaS* strain, at equivalent time point to Y shape formation, was calculated to be 6,195 ± 3,450 μm^2^. At the same time, the average area captured by the same number of wild-type cells, forming the typical Y shape, was 17,387 ± 4,776 μm^2^. Importantly, Y shape formation was resumed when *ltaS* expression was complemented (Figure S2C). Taken together, these results indicate that *ltaS* is essential for the earliest stages of colony establishment. In its absence, the extension of the Y arms is perturbed, resulting in the formation of a small colony.

Since *B. subtilis* possesses three paralogous of *ltaS*: *yfnI, yvgJ*, and *yqgS* (Grundling and Schneewind, 2007; Wormann et al., 2011), we tested whether these genes also have an effect on colony development. All single mutants produced mature colonies similar in size to those of the wild type strain (Figures S3A,B), and exhibited normal early developmental patterns (Figure S3C). Of note, the Δ*ltaS*, Δ*yfnI*, Δy*qgS* triple mutant strain appeared to have a more severe deficiency in colony expansion than the Δ*ltaS* strain (Figure S3C), suggesting that there might be an overlapping function among the paralogous during colony formation. These results are in agreement with previous studies showing that *ltaS* is the major LTA synthase and that single knockouts of each one of the *ltaS* paralogous have no significant effect on cell growth or morphology (Schirner et al., 2009).

### *ltaS* mutant exhibits cell death predominantly at the tips of the extending arms

**Figure 4:**
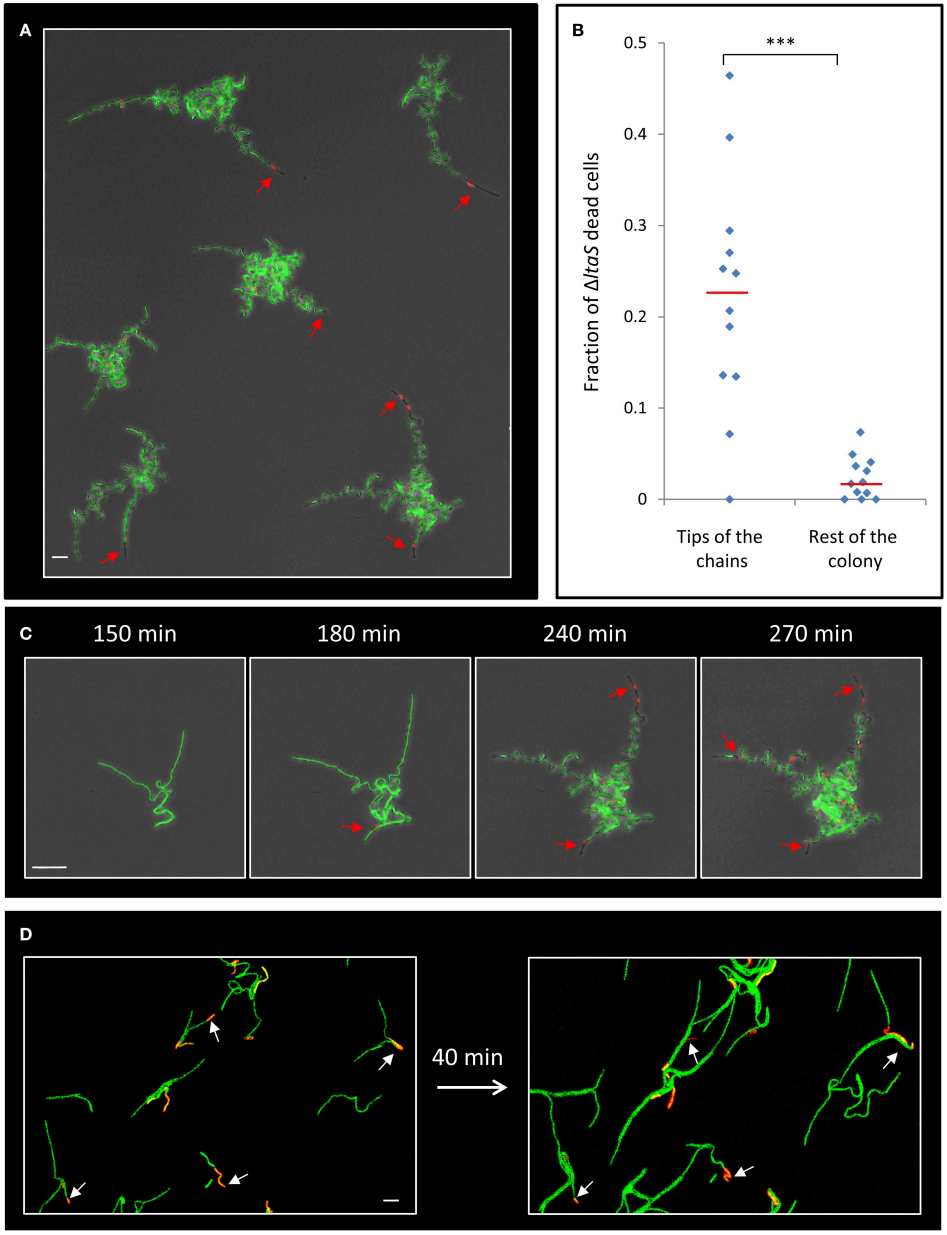
Δ*ltaS* developing colonies exhibit frequent cell death at the tips of the extending arms. **(A)** Colonies of GM24 (Δ*ltaS*, P_*rrnE*_*-gfp*, green) were incubated on LB agar at 37°C in the presence of PI (red) and followed by time lapse microscopy using CLSM. Shown is fluorescence image overlaid by transmitted light image of a large representing field containing several colonies after 5 h of growth. Red arrows highlight dead cells at the tips of the extending arms. Scale bar 10 μm. **(B)** A scatter plot comparing the fraction of dead cells at the tips of the arms with the rest of the colony in the Δ*ltaS* mutant (GM21). Red line represents the average for each category. 12 colonies were quantified in 3 separate experiments. Statistical significance was calculated using unpaired *t*-test. **(C)** Time lapse fluorescence images overlaid by transmitted light images of GM24 (Δ*ltaS*, P_*rrnE*_*-gfp*, green) developing colony grown in the presence of PI (red) and followed by CLSM at the indicated time points. Red arrows highlight the sequential death of all tips. Scale bars 10 μm. **(D)** Fluorescence images of GM24 (Δ*ltaS*, P_*rrnE*_*-gfp*, green) strain grown in the presence of PI (red). Shown is a large representing field containing numerous growing chains on LB agar (Left), and the same field (Right) after 40 min growth at 37°C. Arrows indicate dead cells within the chains. Scale bar 10 μm. ^***^P < 0.001, Very significant.

Next, we attempted to understand the reason underlying the failure of the *ltaS* mutant to create extending arms during the initial stages of colony formation. Close examination of our time lapse microscopy movies hinted at the occasional death of cells within the mutant chains (Movie S3). To follow cell death during early stages of colony development, we supplemented the medium with the red fluorescent stain propidium iodide (PI), which preferentially penetrates damaged or dead cells. Intriguingly, at 4–5 h of development, when the Δ*ltaS* colonies exhibited limited formation of extending arms, dead cells became prevalent, as indicated by the loss of their GFP fluorescence and the accumulation of PI red staining (Figure 4A; Movie S4). Importantly, no such phenomenon was observed for wild type developing colonies grown under similar conditions (Figure S4A). Furthermore, cell death in Δ*ltaS* developing colonies was prominent at the tip of the cell chains (Figures 4A,B), and over time was frequently evident in the tips of all the arms in a given colony (Figure 4C). In general, cell death occurrence seemed abrupt, and usually included the death of a cluster of about 5–10 consecutive cells (Figure S4B). The average fraction of dead cells was calculated to be ~10-fold higher at the chain tips compared to cell death occurring elsewhere in the colony (Figure 4B). Consistent with these findings, examination of cell chains derived from a Δ*ltaS* liquid culture revealed a similar phenomenon, showing frequent cell death at the ends of growing chains (Figure 4D).

**Figure 5:**
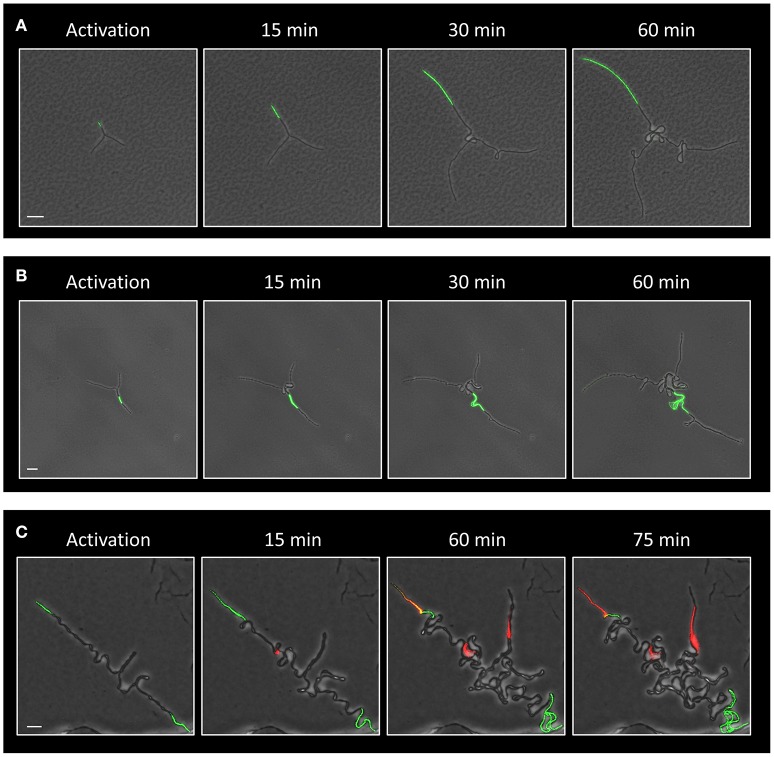
Evidence that Δ*ltaS* colonies fail to expand due to cell death at early stages of development. **(A,B)** Time lapse fluorescence images overlaid by transmitted light images of GB181 (*rplA*-*Dronpa*) developing colonies at the indicated time points followed by CLSM. The Dronpa fluorophore was activated (green) at the tip **(A)** or at the middle **(B)** of the arm, and the irradiated colonies were followed by CLSM. Scale bars 20 μm. **(C)** Time lapse fluorescence images overlaid by transmitted light images of GM41 (Δ*ltaS, rplA*-*Dronpa*) developing colony grown in the presence of PI (red) and followed by CLSM at the indicated time points. The Dronpa fluorophore was activated (green) at the tips of the 2 initial arms. Of note, two arms display PI staining, while the bottom third arm continues to growth without significant extension. Scale bar 10 μm.

We have previously shown that deliberate eradication of cells located at the tips of the Y arms resulted in the formation of a colony with a small diameter, constrained by the limited extension of its arm chains (Mamou et al., 2016). Hence, we reasoned that the frequent death of cells at the ends of the arms in the Δ*ltaS* strain could explain, at least in part, the consequent formation of a small sized colony. To directly examine whether the occasional cell death limits colony size, we devised an approach to track cell lineage within the colony as it develops using a strain expressing the Dronpa photoactivatable protein (Ando et al., 2004). Light activation of Dronpa fluorescently marks the irradiated cell and subsequently its progenies. As a first step, we followed arm extension in colonies derived from a wild type strain. Photoactivation of cells located at the tip of a Y arm resulted in labeling of almost the entire arm by the descendants (Figure 5A; Movie S5). On the other hand, photoactivation of specific cells located at a mid-arm yielded a population of cells located close to the colony center that did not contribute to arm extension (Figure 5B; Movie S6). These results reinforce our previous findings that the cells located at the tip of the Y arm are the major contributors to arm elongation. Next, we followed cell growth using Dronpa, and cell death using PI, in Δ*ltaS* developing colonies. Photoactivation of cells located at the tip of the arms showed initial extension that was frequently followed by cell death of the extending arm (Figure 5C; Movie S7). Notably, even in cases whereby no cell death was monitored at the colony edges, the cell chains curled, creating tangled bundles, only marginally contributing to arm extension (Figure 5C; Figure S4C). Taken together, the inability of Δ*ltaS* developing colonies to create extending arms is mainly due to high frequency of cell death at the arm tip. Loss of directional growth during early stages of development eventually leads to the formation of a small colony.

### LytE, a cell wall hydrolase, affects colony development

**Figure 6:**
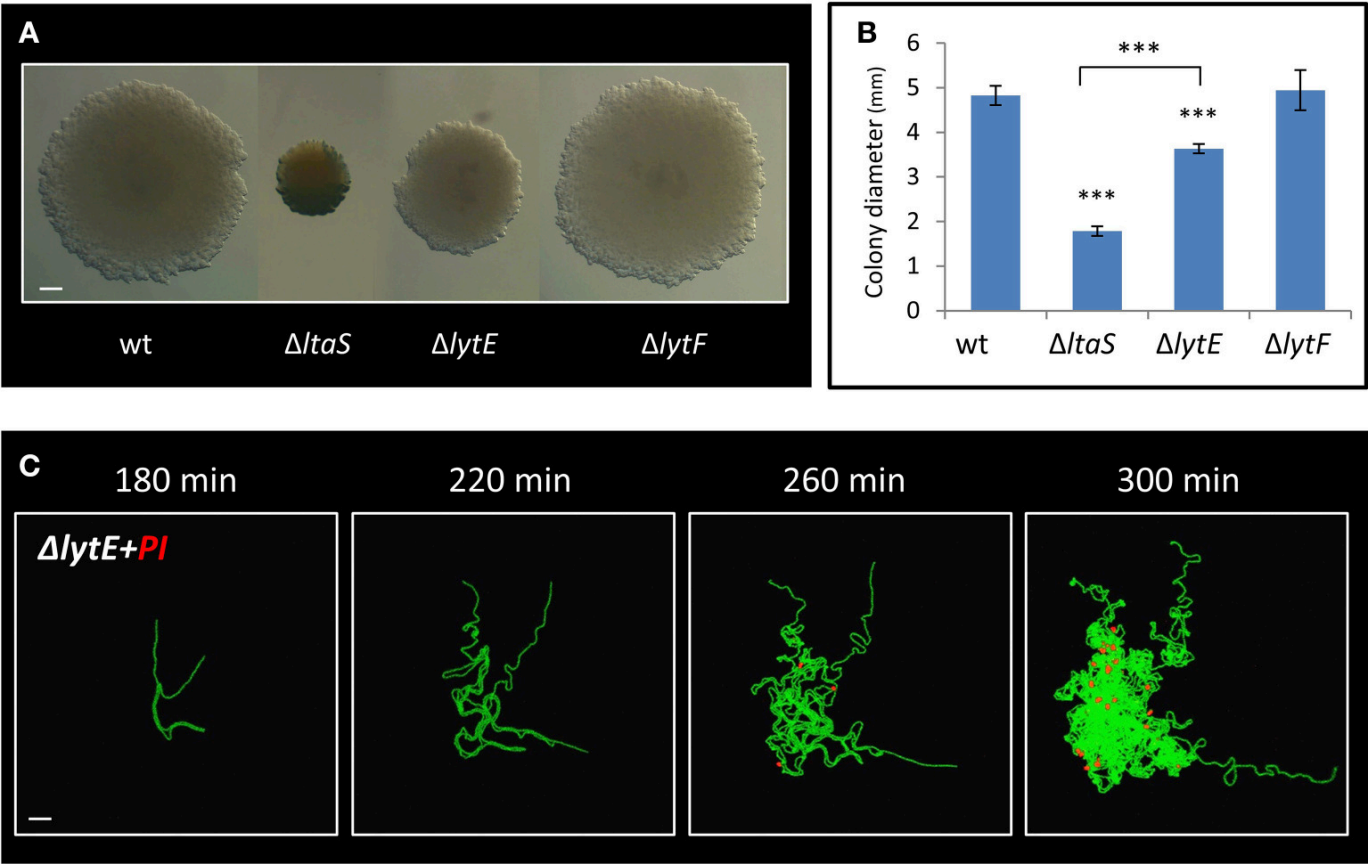
LytE cell wall remodeling enzyme affects early stages of colony development. **(A)** Cells of PY79 (wt), GM21 (Δ*ltaS*), GM22 (Δ*lytF*), and GM37 (Δ*lytE*) were grown on solid LB medium and incubated at 37°C for 20 h. Shown are typical colonies photographed using a binocular. Scale bar 0.5 mm. **(B)** The average diameter of colonies described in A. At least 15 colonies were measured for each strain. Statistical significance was calculated using unpaired *t*-test. **(C)** Time lapse fluorescence images of GM38 (Δ*lytE*, P_*rrnE*_*-gfp*) developing colony followed by CLSM at the indicated time points. PI was added to the medium (red). Scale bar 20 μm. ^***^P < 0.001, Very significant.

LtaS activity was previously shown to be required for proper expression and localization of the LytE and LytF cell wall remodeling enzymes in liquid medium (Kiriyama et al., 2014; Kasahara et al., 2016). *lytF*, was found to be underexpressed in Δ*ltaS* cells (Kiriyama et al., 2014), yet deleting the gene had no significant effect on colony size or Y shape formation (Figures 6A,B; Figure S5A). On the other hand, *lytE* was shown to be overexpressed in the absence of *ltaS* (Kasahara et al., 2016). We therefore examined whether deletion of *lytE* can suppress the Δ*ltaS* phenotype. However, the Δ*lytE* Δ*ltaS* double mutant strain produced colonies, which were even smaller than those of the Δ*ltaS* strain (Figures S5B,C). These findings suggest that Δ*ltaS* deficiency in forming colonies is not simply a result of modified expression of neither *lytF* nor *lytE*. Examining the effect of Δ*lytE* single mutation on colony size revealed that Δ*lytE* cells produced colonies that were visibly smaller than wild type, but larger than Δ*ltaS* (Figures 6A,B). Microscopic inspection indicated that the Δ*lytE* strain produced typical Y shapes, but frequently failed to progress through the subsequent stages of arm extension and central thickening (Figure S5D; Movie S8). Like Δ*ltaS* cells, chains of Δ*lytE* curled and coiled instead of expanding outwards from the colony midpoint.

We next examined whether, similarly to Δ*ltaS*, dead cells accumulate in the Δ*lytE* colony as it develops. As indicated by PI staining, dead cells started to appear ~4 h after colony initiation, at a time corresponding to their emergence in Δ*ltaS* colonies (Figure 6C). Furthermore, the number of dead cells within the Δ*lytE* colony clearly increased over time (Figure 6C). Importantly, no cell death was detected in wild type colonies at equivalent time points (Figure S4A). In contrast to the Δ*ltaS* mutant, the dead cells were located within the bulk of the Δ*lytE* colony, and were mostly absent from the arm tips (Figure 6C; Figure S5E). No cell death was observed in chains of Δ*lytE* grown in liquid, as opposed to the chains of the Δ*ltaS* strain (Figure 4D; Figure S5F). Hence, both mutants are deficient in colony development and exhibit high frequency of cell death. However, the cell death pattern of the mutant colonies is dissimilar, with Δ*ltaS* displaying more severe deficiency due to enhanced cell death at the leading edge.

## Discussion

We revealed that LtaS, the major LTA synthase, is required for proper execution of the earliest stages of colony development, thereby producing small sized colonies. LtaS mutant cells are deficient in cell chain breakage, forming continuous “endless” chains, and thus perturbed in forming the typical Y shape structure. A key finding in understanding how LtaS mutation restricts colony size is the observation of frequent cell death at the tips of the cell chains initiating the Δ*ltaS* colony. This distinctive cell death has a crucial impact on colony expansion, as it limits the reach of the chains that determines the boundaries and the size of the future colony. Taken together, it appears that the damage to the cell envelope, due to the lack of LTA, causes a mechanical failure, impeding the proper execution of the colony developmental program.

LtaS is a membrane protein with several transmembrane helices connected to a large extracellular domain, which accommodates the active site required for glycerol-phosphate polymerization. Furthermore, LtaS from *Staphylococcus aureus* (*S. aureus*) was shown to be sufficient to induce LTA synthesis in *E. coli*, lacking LTA (Grundling and Schneewind, 2007; Lu et al., 2009). Although in Gram positive bacteria LTA polymers comprise a significant proportion of the cell envelope and play an important role in bacterial growth and physiology, the actual function of LTA remains elusive (Percy and Grundling, 2014; Schneewind and Missiakas, 2016). LTAs of *B. subtilis* are lipid anchored poly glycerol-phosphate chains, modified with D-alanine and N-acetylglucosamine (Villeger et al., 2014). The integrity of LTA was shown to be required for proper cell morphology and division, as LtaS mutant exhibited defects in septum formation and cell separation (Schirner et al., 2009). Previous studies have shown that only *ltaS*, but not its paralogues, is capable of functionally complementing the lack of *S. aureus* LTA synthase (Grundling and Schneewind, 2007). Furthermore, no significant defects in cell division and morphology were observed for mutants in the *ltaS* paralogous (Schirner et al., 2009; Kiriyama et al., 2014). These findings are in accord with our current results, showing that mutating each paralogous does not affect colony development. Interestingly, in addition to a role in cell division, LtaS from *S. aureus* was found to be crucial for proper cell envelope assembly, and was suggested to act as an osmoprotectant maintaining cellular integrity (Oku et al., 2009; Percy and Grundling, 2014). This latter finding can be linked to our current observation that LtaS mutant cells frequently lose their integrity, becoming permeable to PI.

One of the most interesting questions is why the cells at the tip of the LtaS mutant chains tend to perish more frequently than the rest of the cells in the colony. Intriguingly, simple analysis of the colony descendants predicts that the cells at the tip of the extending chains are the oldest cells in the colony (Figure S6), signifying that aging leads to cell death. Accumulating evidence indicates that bacterial cells exhibit apparent aging-associated phenotypes, such as accumulation of defective proteins and inclusion bodies (Lindner et al., 2008; Rokney et al., 2009; Winkler et al., 2010). Furthermore, following aging in *E. coli* cells revealed that after ~50 divisions, cells tend to produce filaments, a clear sign of stress (Wang et al., 2010). In this regard, we have shown that the cells located at the tip of the extending chains are engaged in extensive cell divisions, suggesting that they are prompt to age rapidly. Since in our experimental design cell death was not apparent in wild type developing colonies, it seems that the lack of LTA enforces pre-mature cell aging. The accelerated aging could be caused by the deficiency of the mutant cells to face osmotic stress, as was implicated for the LTA mutant from *S. aureus*, whereby LTA was shown to strengthen cell envelope and provide rigidity to the cell surface, features that can be crucial during cell aging (Oku et al., 2009; Schneewind and Missiakas, 2016). In line with this view, cells located at the chain ends display a larger exposed surface area, in comparison to the other cells in the chain, a fact that can contribute to increased osmotic stress and rapid surface rupturing.

While our results indicate that mutating *ltaS* directly affects colony development, the mutation seems to influence additional genes impacting the process. The proper expression and localization of the cell wall hydrolase LytE was shown previously to be dependent on LtaS (Kasahara et al., 2016). Our inspection revealed that LytE plays a role in proper colony development mainly by influencing Y arm extension and colony thickening patterns, and by exhibiting increased cell death throughout the colony, phenotypes that are markedly different from those observed for the *ltaS* mutant. Nevertheless, it is possible that the *ltaS* mutant phenotype is multifactorial, inflicted by the cell response to envelope stress caused by the lack of LTA (Hashimoto et al., 2013).

In this study, we show that the formation of small colonies in *B. subtilis* is not merely a consequence of growth defects, but emanates from a failure to execute early developmental stages occurring hours prior to the establishment of the mature colony. Further, our data reinforce the view that the reach of the cell chains determines colony expansion. We have previously shown that a phosphodiesterase YmdB affects the trajectory patterns of the Y arms, playing a regulatory role in guiding these initial events (Diethmaier et al., 2014; Mamou et al., 2016). YmdB impact on colony formation was shown to be linked to intercellular cAMP levels and the ability of the Y arms to form intercellular nanotubes to coordinate their growth (Mamou et al., 2016). Here we show that mechanical perturbations, the lack of timed chain breakage as well as accelerated cell rupturing, affect the pattern of arm extension and thereby the ultimate colony size.

## Author contributions

GM designed the experiments, conducted the research and wrote the manuscript. OF designed the experiments and conducted the research. LS Conducted the genetic screen. SB Supervised the project, designed, and analyzed the experiments and wrote the manuscript.

### Conflict of interest statement

The authors declare that the research was conducted in the absence of any commercial or financial relationships that could be construed as a potential conflict of interest.
