# Supplementary material for: Deficiency in Lipoteichoic Acid Synthesis Causes a Failure in Executing the Colony Developmental Program in Bacillus subtilis
Source: Front Microbiol. 2017 Oct 24;8:1991. doi: 10.3389/fmicb.2017.01991 (PMC5660684; doi:10.3389/fmicb.2017.01991)

Supplementary Material

Deficiency in lipoteichoic acid synthesis causes a failure in executing the colony developmental program in *Bacillus subtilis*

Gideon Mamou[1], Osher Fiyaksel[1], Lior Sinai[1] and Sigal Ben-Yehuda[1,2]

*** Correspondence:** Corresponding Author: sigalb@ekmd.huji.ac.il

Table S1: List of bacterial strains used in this study

Strain Name	Genotype	Description
PY79	Wild type	(Youngman et al., 1984)
4620	*trpC2 ΔltaS::neo, ΔyfnI::cat, ΔyqgS::spc, ΔyvgJ::erm*	A gift from J. Errington Newcastle University (Schirner et al., 2009).
AR16	*amyE::P*$_{rrnE}$*-gfp-spc*	(Rosenberg et al., 2012)
GB181	*rplA-Dronpa-spec*	Constructed by transforming pGB17 into PY79.
GM21	Δ*ltaS::mls*	Constructed using Gibson assembly kit (NEB, USA) utilizing primers *ltaS*-ko-P1-P4.
GM22	Δ*lytF::cm*	Constructed using Gibson assembly kit (NEB, USA) utilizing primers *lytF*-ko-P1-P4.
GM24	Δ*ltaS::mls , amyE::PrrnE-gfp-spc*	Constructed by transforming AR16 gDNA into GM21.
GM27	Δ*yfnI::cm*	Constructed using Gibson assembly kit (NEB, USA) utilizing primers *yfnI*-ko-P1-P4.
GM37	Δ*lytE::Kan*	Constructed using Gibson assembly kit (NEB, USA) utilizing primers *lytE*-ko-P2-P6.
GM38	Δ*lytE::Kan , amyE::*P$_{rrnE}$*-gfp-spc*	Constructed by transforming AR16 gDNA into GM37.
GM39	Δ*lytF::Kan*	Constructed using Gibson assembly kit (NEB, USA) utilizing primers *lytF*-ko-P2,P5 and P3,P6.
GM40	Δ*lytf::Kan , amyE::*P$_{rrnE}$*-gfp-spc*	Constructed by transforming AR16 gDNA into GM39.

GM41	Δ*ltaS::mls , rplA-Dronpa-spec*	Constructed by transforming GB181 gDNA into GM21.
GM42	Δ*ltaS::mls*, Δ*lytE::Kan*	Constructed by transforming GM21 gDNA into GM37.
GM45	Δ*ltaS::mls*, *amyE::*$P_{hyperspank}$-*ltaS-spc*	Constructed by transforming pGM14 into GM21.
GM52	Δ*yqgS::spc*	Constructed by transforming 4620 gDNA into PY79.
GM54	Δ*ltaS::mls*, Δ*yqgS::spc*	Constructed by transforming 4620 gDNA into GM21.
GM57	Δ*yvgJ::mls*	Constructed by transforming 4620 gDNA into PY79.
GM58	Δ*ltaS::mls*, Δ*yqgS::spc*, Δ*yfnI::cm*	Constructed by transforming 4620 gDNA into GM54.

B. subtilis strains are derivatives of the wild type strain PY79 (Youngman et al., 1984).

Table 2: List of plasmids used in this study

Plasmid	Genotype	Description
pGB27	*rplA-Dronpa-spec*	(Dubey et al., 2016).
PGM14	*amyE*::$P_{hyperspank}$-*ltaS-spec*	Constructed by amplifying the *ltaS* ORF from genomic DNA of wild type *B. subtilis* strain (PY79), using primers *ltaS*-U-*NheI* and *ltaS*-l-*SphI* The PCR-amplified DNA was digested with *Nhe*I and *Sph*I and was cloned into pDR111 digested with the same enzymes.

Plasmid constructions were performed in DH5α *E. coli* cells.

Table 3: List of primers used in this study

Primer Name	Primer Sequence (5'-3')
ltaS- ko –P1	ATAAGCTCTTTTTTGAGCGCG
ltaS- ko –P2	CTGAGCGAGGGAGCAGAATGTTACACTCCTTTTTTCCGATC
ltaS- ko –P3	GTTGACCAGTGCTCCCTGTAAGAAAAAGCGGAGAGGTTGC
ltaS- ko –P4	CGGTCTTAACCGGGTTATCTTT
ltaS-U-NheI	AAACCCGCTAGCAAAGGTGGTGAACTACTATGAAAACATTTATAA AAGAAAGAGGACTG
ltaS-l-SphI	ACCTAGGCATGCTTATTATTATTTATCTTCGTTATCCTTTGACGTTT
lytE- ko –P2	CTGAGCGAGGGAGCAGAAATTTTCCTCCCCAAATGTTAACTCTATA TATATGTA
lytE- ko –P3	GTTGACCAGTGCTCCCTGTAATTTTTAGAGAAAACCCGTTCATTGG AA
lytE- ko –P5	AACAATAATGAAAGCCTTGTAAGGAAATG
lytE- ko –P6	CAGTCTTCTTCTTTAAGCTGCC
lytF- ko –P1	AAAGTCATATACAGGCTCGGC
lytF- ko –P2	CTGAGCGAGGGAGCAGAACTTAGTTCTCCTTTTTCCCCC

lytF- ko –P3	GTTGACCAGTGCTCCCTGTAAAAACAGAAACTGTGCGGC
lytF- ko –P4	GAGGGAGACATAAAATCAAAATGCC
lytF- ko –P5	ACAGATTGCCGAAACGTATTC
lytF- ko –P6	ATGCCGCATCCTCTGTTATT
yfnI–ko –P1	AGGGATATAGATAAAGAAGACTGGAGA
yfnI –ko –P2	CTGAGCGAGGGAGCAGAAGGCAACCTCTACTTTCTTAAACAC
yfnI –ko –P3	GTTGACCAGTGCTCCCTGTTAAGATGAAAAAGAGCCTTGAGCG
yfnI –ko –P4	TCTCCAAAAACGTAATTTTCCACGTT

Supplementary Figure Legends

Figure S1: Transposon mutants identified in the genetic screen for deficiency in colony formation

(A) A table presenting transposon mutants which were found to produce small or aberrant shaped colonies in the transposon screen. The name and annotation (http://subtiwiki.uni-goettingen.de/) of the disrupted gene are listed. Images represent colonies of the indicated mutant colonies after 24 hrs of incubation on LB agar at 37°C. Scale bar 0.5 mm.

(B) Growth curves of wt (PY79) and the indicated transposon mutant strains in liquid LB at 37°C.

Figure S2: Characterization of the *ΔltaS* mutant

(A) Percentage of initial GM21 (*ΔltaS*) colony morphologies exhibiting normal Y shape in comparison to PY79 (wt). For each strain ~50 developing colonies were examined.

(B) Growth curves of PY79 (wt) and GM21 (*ΔltaS*) strains in liquid LB at three different temperatures.

(C) Time lapse transmitted light images of GM45 (*ΔltaS, $P_{hyper\text{-}spank}$- ltaS*) developing colony followed by light microscopy at the indicated time points. Scale bar 25 µm.

Figure S3: *ltaS* paralogous have minor effect on colony development

(A) Cells of the wt (PY79) and indicated mutant strains were grown on solid LB medium and incubated at 37°C for 20 hrs. Shown are typical colonies photographed using a binocular. Notice that although the colony size of the *ΔltaS* and the triple mutant strains was similar, the texture was different with the triple mutant producing a very round and smooth edged colony. Scale bar 0.5 mm.

(B) The average diameter of colonies described in A. At least 10 colonies were measured for each strain. Statistical significance was calculated using unpaired t-test.

(C) Colonies of wt (PY79) and the indicated mutant strains were incubated on LB agar at 37°C and followed by time lapse microscopy. Shown are developing colony images taken at 3 and 4.5 hrs of growth. Scale bar 20 µm.

Figure S4: Characterizing the pattern of cell death in *ΔltaS* mutant.

(A) Time lapse transmitted light images of wt (PY79) developing colony grown in the presence of PI and followed by light microscopy at the indicated time points. Scale bar 20 µm.

(B) Upper panel: Time lapse fluorescence images overlaid by transmitted light images of GM24 (*ΔltaS*, P_{rrnE}-*gfp,* green) developing colony grown in the presence of PI (red) and followed by CLSM at the indicated time points. Lower panel: A magnification of the bottom arm's tip where the dead cells emerge. Scale bars 10 µm

(C) Time lapse fluorescence images overlaid by transmitted light images of GM41 (*ΔltaS ,rplA-Dronpa*) cells at the indicated time points during colony formation. The Dronpa fluorophore was activated at the tip of the arm and colony development was followed. Scale bar 10 µm.

Figure S5: LytE has diverse effects on colony development

(A) Time lapse fluorescence images of GM40 (*ΔlytF*, P_{rrnE}-*gfp*) developing colony followed by CLSM at the indicated time points. Scale bar 20 µm.

(B) Cells of PY79 (wt), GM21 (*ΔltaS*) and GM42 (*ΔltaS ΔlytE*) were grown on solid LB medium and incubated at 37°C for 20 hrs. Shown are typical colonies photographed using a binocular. Scale bar 0.5 mm.

(C) The average diameter of colonies described in A. At least 10 colonies were measured for each strain. Statistical significance was calculated using unpaired t-test.
(D) Time lapse fluorescence images of GM38 (*ΔlytE*, P_{rrnE}-*gfp*) developing colony followed by CLSM at the indicated time points. Scale bar 20 µm.
(E) A scatter plot comparing the fraction of dead cells at the tips of the arms with rest of the colony in the *ΔlytE* mutant (GM38). Red line represents the average for each category. 11 colonies were quantified.
(F) Fluorescence images of GM24 (*ΔlytE*, P_{rrnE}-*gfp*, green) strain grown in the presence of PI (red). Shown is a large representing field containing numerous cell chains grown on LB agar (Left), and the same field (Right) after 30 min growth at 37°C. No dead cells were monitored within the chains. Scale bar 10µm.

Figure S6: A model for cell aging directing cell death in cell chains of *ΔltaS*
We propose that the cells at the tip of chains of developing colonies are the oldest ones, containing the pole originating from the progenitor cell. Accordingly, the cell in grey is the progenitor cell that divides to create a chain with its original poles located at both cell ends. In Δ*ltaS*, PI penetrates the cells with the oldest poles located at the tips. The death of the cells at the tips limits colony expansion.

Supplementary Movie legends:

Movie S1: Δ*ltaS* colony fails to exhibit typical developmental patterns
Time lapse images of Δ*ltaS* colony formation. Cells of GM24 (*ΔltaS*, P_{rrnE}-*gfp*) were grown on solid LB at 37°C. Images were taken by CLSM at 20 min intervals in the course of 3 hrs.

Movie S2: Wild-type colony exhibits the typical early stages of colony development
Time lapse images of wild type colony formation. Cells of AR16 (P_{rrnE}-*gfp*) were grown on solid LB at 37°C. Images were taken by CLSM at 20 min intervals in the course of 3 hrs.

Movie S3: Occasional death of cells within a Δ*ltaS* developing colony
Time lapse images of Δ*ltaS* colony formation. Cells of GM21 (*ΔltaS*) were grown on solid LB at 37°C. Phase contrast images were taken at 10 min intervals in the course of 2.5 hrs. Red arrows indicate sites of cell death.

Movie S4: Δ*ltaS* colonies exhibit frequent cell death at the tip of the arms
Time lapse images of Δ*ltaS* colony formation in the presence of PI (Red). Cells of GM24 (*ΔltaS*, P_{rrnE}-*gfp*) (Green) were grown on solid LB at 37°C. Fluorescence and phase contrast images were taken by CLSM at 20 min intervals in the course of 2.5 hrs.

Movie S5: Cells at the tip of the chain propel arm extension
Time lapse images of colony growth, with Dronpa marking the cells at the tip of the Y arm (Green). Cells expressing RplA-Dronpa (GB181) were grown on solid LB at 37°C. The Dronpa fluorophore was activated only in the cells at the tip of the arm. Fluorescence and phase contrast images were taken by CLSM at 20 min intervals in the course of 2 hrs.

Movie S6: Cells at the mid-arm grow towards the colony centre as the arm extends
Time lapse images of colony growth with Dronpa marking the cells at the middle of the Y arm (Green). Cells expressing RplA-Dronpa (GB181) were grown on solid LB at 37°C. The Dronpa fluorophore was activated only in the cells at the middle of the double chained arm. Fluorescence and phase contrast images were taken by CLSM at 15 min intervals in the course of 2 hrs.

Movie S7: Cells at the tip of the arm fail to extend and subsequently perish
Time lapse images of colony growth with Dronpa marking of the cells at the tip of the Y arm (Green) in the presence of PI (Red). Cells of GM41 (*ΔltaS* ,rplA-Dronpa) were grown on solid LB at 37°C. Fluorescence and phase contrast images were taken by CLSM at 15 min intervals in the course of 2.5 hrs.

Movie S8: *ΔlytE* developing colony produces a Y-shape but then fails to extend
Time lapse images of Δ*lytE* colony formation. Cells of GM24 (*ΔltaS*, P_{rrnE}-*gfp*) were grown on solid LB at 37°C. Phase contrast and fluorescence images were taken by CLSM at 15 min intervals in the course of 3 hrs.

Supplementary references

Dubey, G.P., Malli Mohan, G.B., Dubrovsky, A., Amen, T., Tsipshtein, S., Rouvinski, A., Rosenberg, A., Kaganovich, D., Sherman, E., Medalia, O., and Ben-Yehuda, S. (2016). Architecture and Characteristics of Bacterial Nanotubes. *Dev Cell* 36, 453-461.

Rosenberg, A., Sinai, L., Smith, Y., and Ben-Yehuda, S. (2012). Dynamic expression of the translational machinery during *Bacillus subtilis* life cycle at a single cell level. *PLoS One* 7, e41921.

Schirner, K., Marles-Wright, J., Lewis, R.J., and Errington, J. (2009). Distinct and essential morphogenic functions for wall- and lipo-teichoic acids in *Bacillus subtilis*. *EMBO J* 28, 830-842.

Youngman, P., Perkins, J.B., and Losick, R. (1984). Construction of a cloning site near one end of Tn917 into which foreign DNA may be inserted without affecting transposition in *Bacillus subtilis* or expression of the transposon-borne *erm* gene. *Plasmid* 12, 1-9.

Figure S1

A

**PY79**	wt	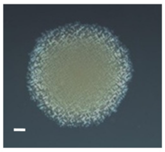
**General machinery**		
clpP	Clp protease proteolytic subunit	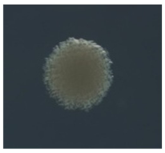
rnhC	Endoribonuclease	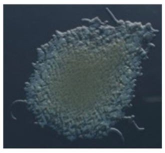
secE	Preprotein translocase subunit	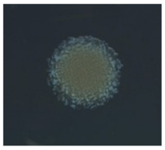
dnaN	Beta clamp, DNA polymerase III	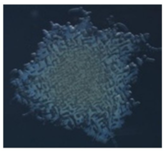
atpG	ATP synthase	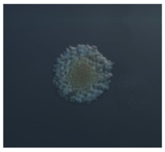

**Multicellularity**		
ecsB	ABC transporter	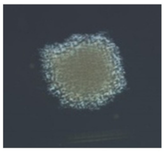
sdpC	Toxin, kills non-sporulating cells	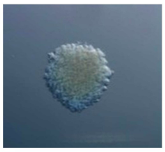
sdpI	Protection against SdpC toxin	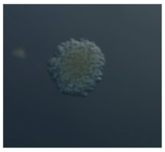
comQ	Regulation of quorum sensing	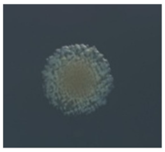
**Stress response**		
yycL	Control of DegU activity	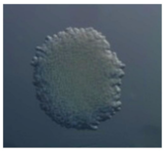
yhdL	Anti-SigM protein	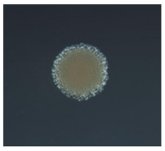

B

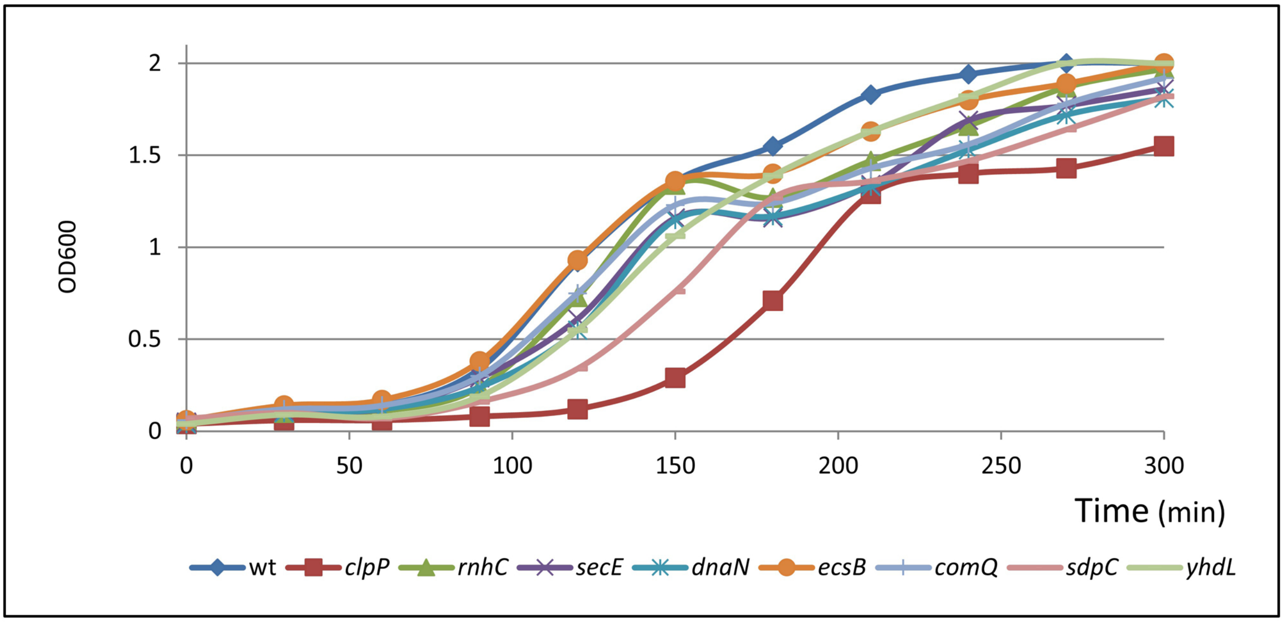

Figure S2

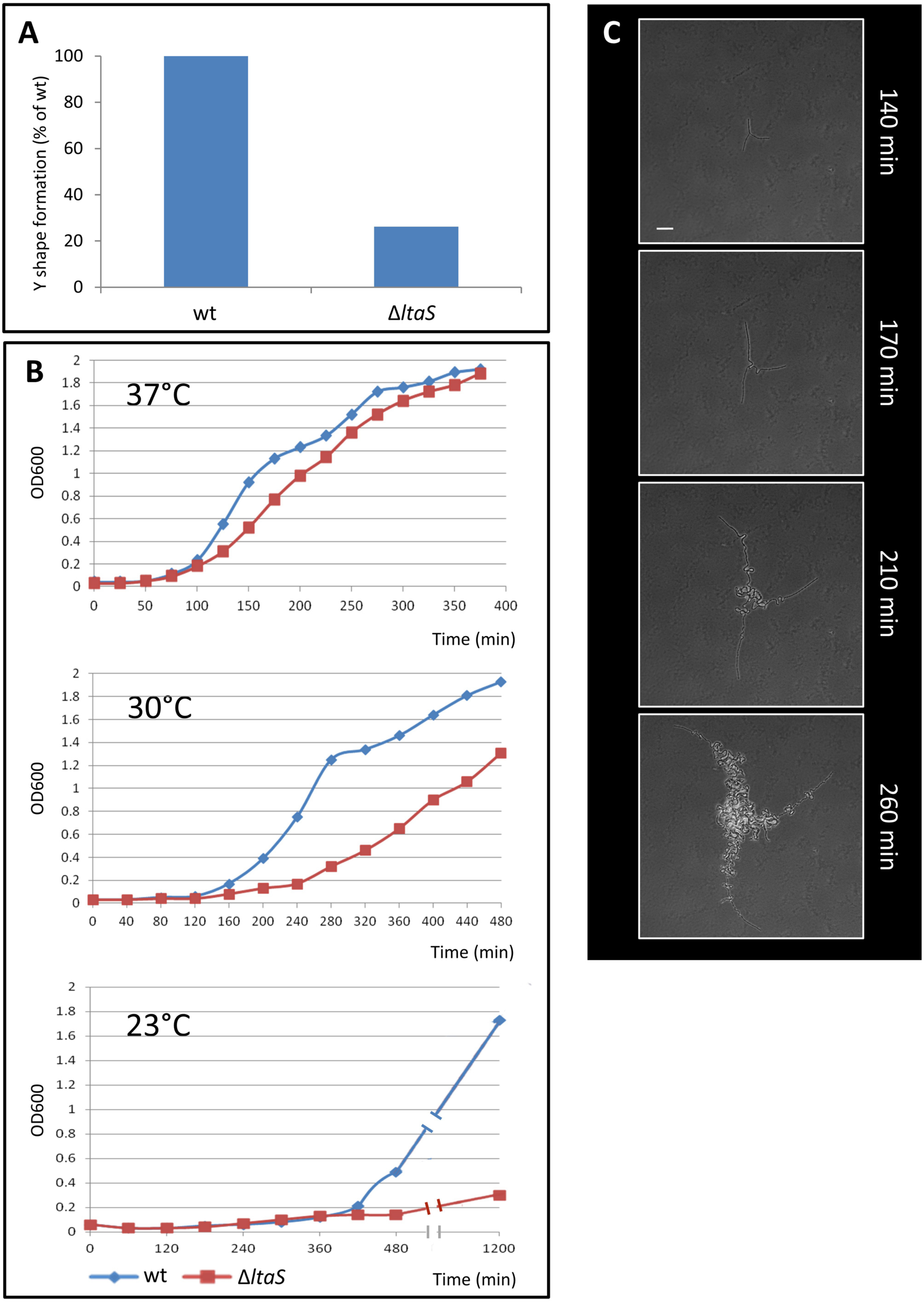
A
Y shape formation (% of wt)
100
80
60
40
20
0
wt
ΔltaS
B
37°C
30°C
23°C
OD600
Time (min)
wt
ΔltaS
C
140 min
170 min
210 min
260 min

Figure S3

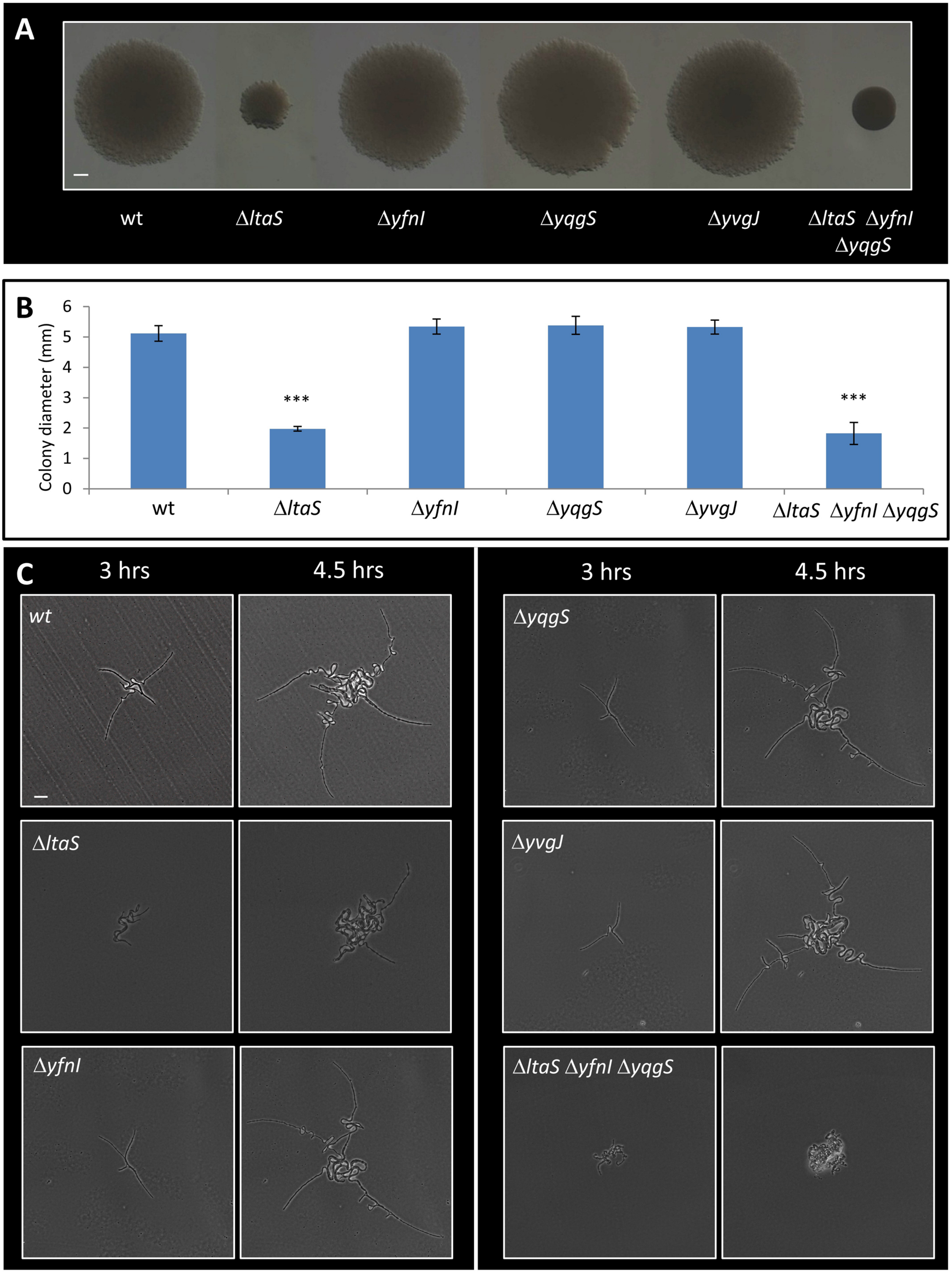
A
wt
ΔltaS
ΔyfnI
ΔyqgS
ΔyvgJ
ΔltaS ΔyfnI ΔyqgS
B
Colony diameter (mm)
0
1
2
3
4
5
6
***
***
wt
ΔltaS
ΔyfnI
ΔyqgS
ΔyvgJ
ΔltaS ΔyfnI ΔyqgS
C
3 hrs
4.5 hrs
wt
ΔltaS
ΔyfnI
3 hrs
4.5 hrs
ΔyqgS
ΔyvgJ
ΔltaS ΔyfnI ΔyqgS

Figure S4

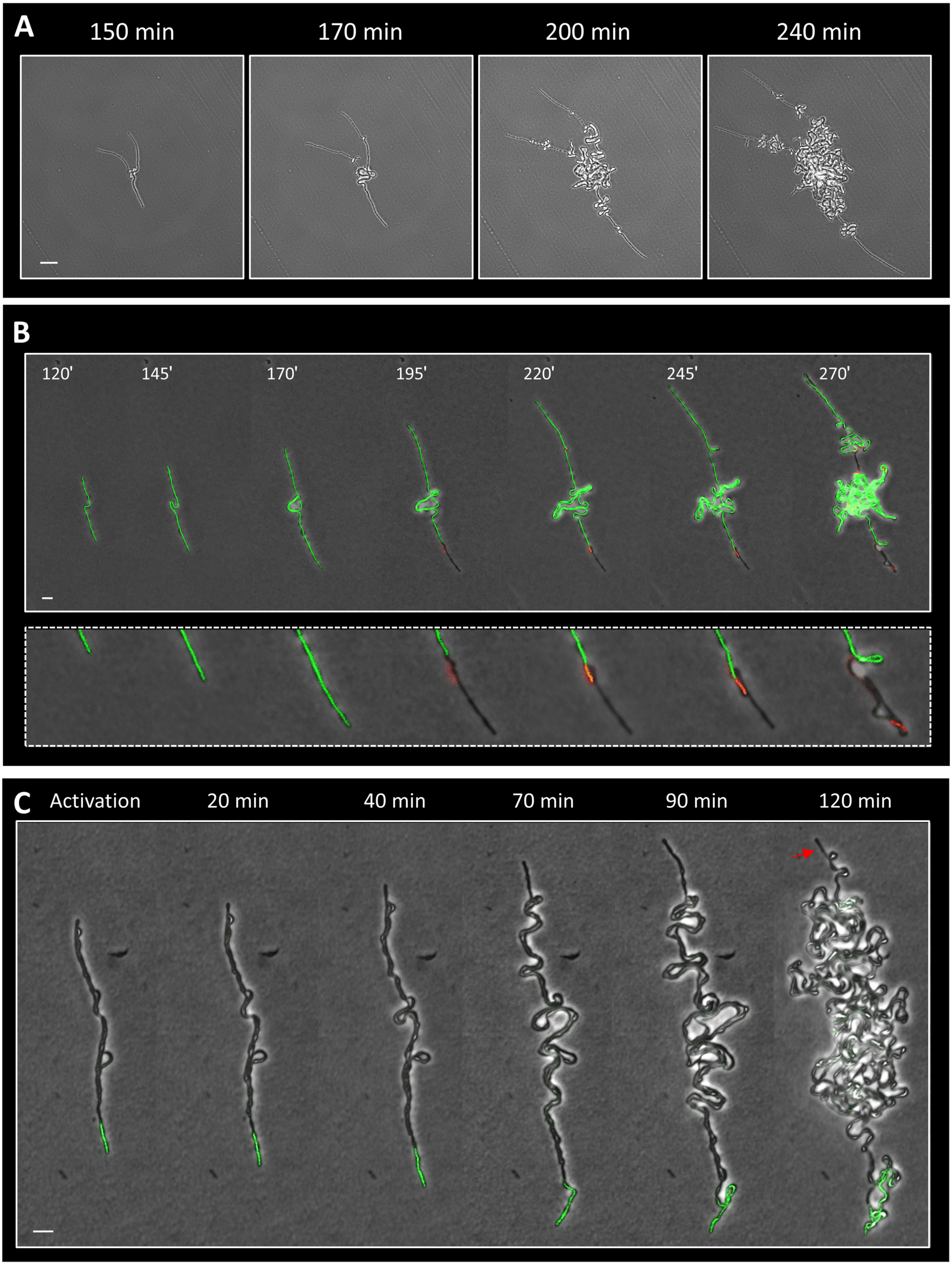
A
150 min
170 min
200 min
240 min
B
120'
145'
170'
195'
220'
245'
270'
C
Activation
20 min
40 min
70 min
90 min
120 min

Figure S5

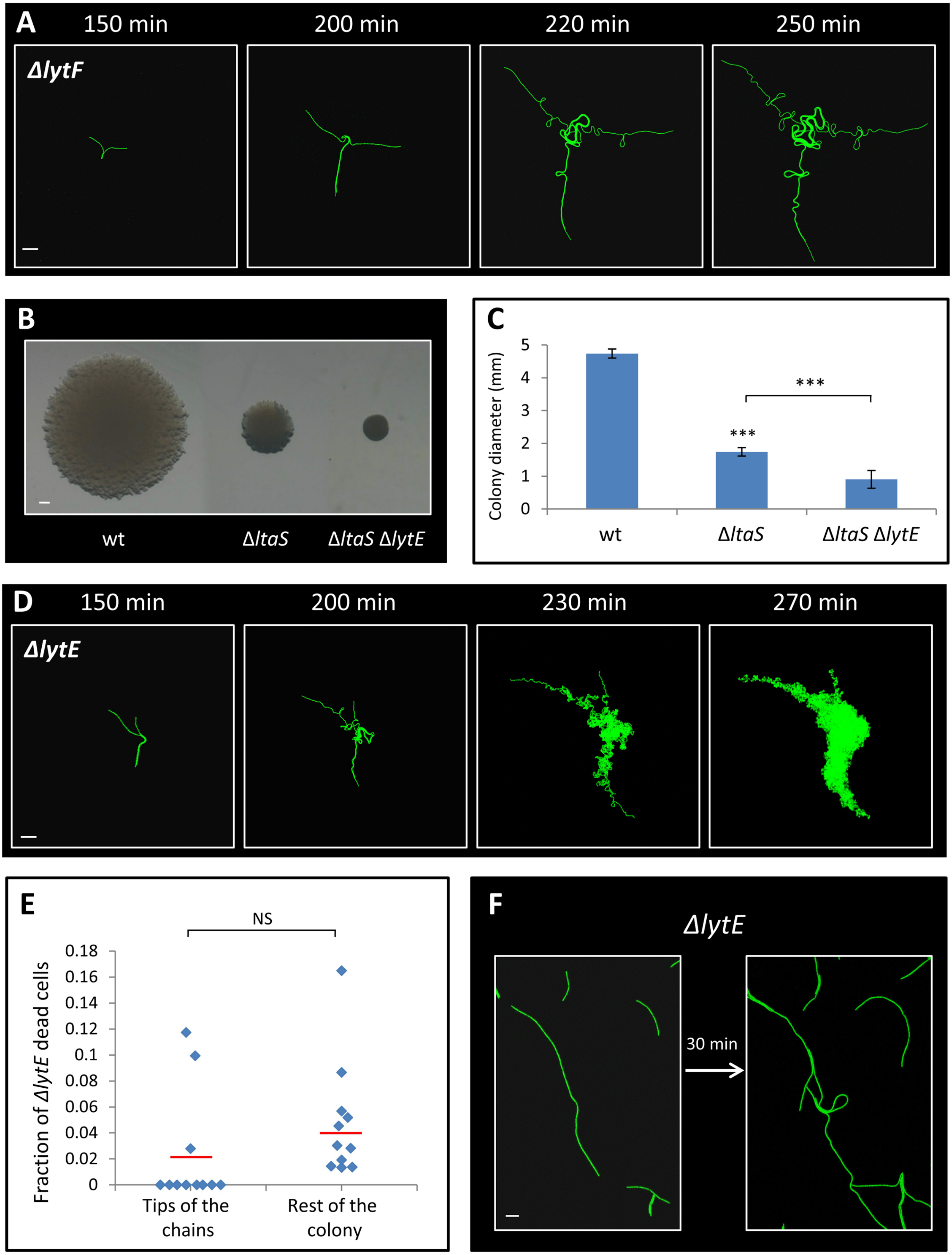
A
150 min
200 min
220 min
250 min
ΔlytF
B
wt
ΔltaS
ΔltaS ΔlytE
C
Colony diameter (mm)
5
4
3
2
1
0
***
***
wt
ΔltaS
ΔltaS ΔlytE
D
150 min
200 min
230 min
270 min
ΔlytE
E
NS
Fraction of ΔlytE dead cells
0.18
0.16
0.14
0.12
0.1
0.08
0.06
0.04
0.02
0
Tips of the chains
Rest of the colony
F
ΔlytE
30 min

Figure S6

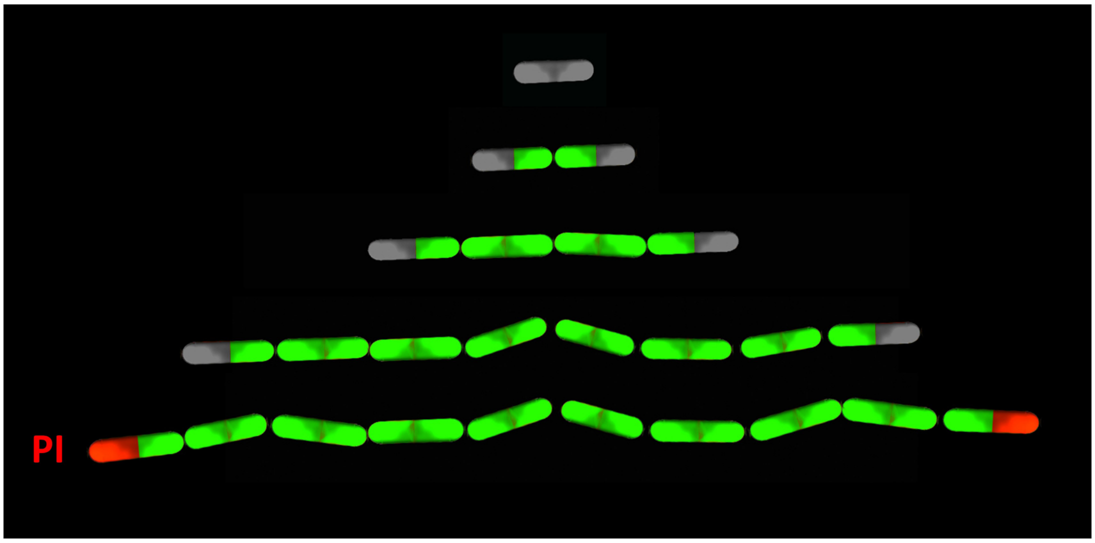

Supplement: Supplementary file 9 [file DataSheet1.PDF]
